# Effect of Multi-Phosphonate Coating of Titanium Surfaces on Osteogenic Potential

**DOI:** 10.3390/ma13245777

**Published:** 2020-12-17

**Authors:** Christian Wehner, Christian Behm, Selma Husejnagic, Andreas Moritz, Xiaohui Rausch-Fan, Oleh Andrukhov

**Affiliations:** 1 Division of Conservative Dentistry and Periodontology, University Clinic of Dentistry, Medical University of Vienna, 1090 Vienna, Austria; christian.wehner@meduniwien.ac.at (C.W.); selma.husejnagic@meduniwien.ac.at (S.H.); andreas.moritz@meduniwien.ac.at (A.M.); xiaohui.rausch-fan@meduniwien.ac.at (X.R.-F.); 2 Competence Center for Periodontal Research, University Clinic of Dentistry, Medical University of Vienna, 1090 Vienna, Austria; christian.behm@meduniwien.ac.at; 3 Division of Orthodontics, University Clinic of Dentistry, Medical University of Vienna, 1090 Vienna, Austria

**Keywords:** implant titanium surface, surface modification, multi-phosphonate coating, osseointegration, osteoblast-like cells, mesenchymal stem cells, periodontal ligament stem cells

## Abstract

The aim of this study was to evaluate the impact of a novel multi-phosphonate (MP) coating strategy of dental implant surfaces on the expression of osteogenesis-related factors in vitro. MG-63 human osteoblast-like cells, bone marrow mesenchymal stem cells (BM-MSCs), and human periodontal ligament stem cells (hPDLSCs) were cultured separately on titanium disks with and without MP coating. Cell attachment was visualized by focal adhesion and actin cytoskeleton staining. The proliferation and gene expression of the markers related to osteogenesis and bone turnover were measured after 48 and 120 h of cell culture. Actin cytoskeleton assembly and focal adhesion were similar between test surfaces within each cell type but differed from those on tissue culture plastic (TCP). The proliferation of MG-63 cells and PDLSCs was comparable on all surfaces, while BM-MSCs showed an increase on tissue culture plastic (TCP) versus titanium. The gene expression of osteoprotegerin and receptor activator of nuclear factor-kappa B ligand was higher in MG-63 cells grown on MP-coated surfaces. At the same time, osteocalcin was decreased compared to the other surfaces. Collagen type I gene expression after 120 h was significantly lower in hPDLSCs cultivated on MP-coated surfaces. Within the limitations of this study, MP coating on titanium surfaces might have a slight beneficial effect on bone turnover in vitro.

## 1. Introduction

The osseointegration process of dental implants is a key requirement for clinical success and is affected by surface characteristics such as roughness, wettability, and chemical composition [1,2]. For decades, titanium has been used frequently not only in implantology but also in other dental fields. This material is characterized by favorable properties such as high biocompatibility and resistance towards fracture and corrosion [3]. Metal alloys such as nickel-titanium (NiTi) have shown good mechanical and clinical properties as material for orthodontic archwires [4]. In endodontic applications, NiTi files exhibit high fatigue tolerance and flexibility, reducing the risk of file breaking inside the root canal [5]. Among various concepts for surface modifications that intend to improve osseointegration, strategies using multi-phosphonate (MP) coating have shown promising results [6,7]. Phosphonates are biologically active phosphate analogs, characterized by a substitution of a carbon atom instead of an oxygen atom, thus increasing the stability against enzymatic hydrolysis of the molecule [8]. When used as a surface coating, phosphonates have been demonstrated to enhance surface hydrophilicity [9]. This suggests beneficial effects for the healing process following implant insertion, as hydrophilic surfaces exhibit improved adsorption of proteins and enhanced angiogenesis during early implant osseointegration [10,11]. 

Coating the implant body with MP molecules generates a surface imitating natural hydroxyapatite (HA), which is the main inorganic constituent of bone [7,12]. Due to its favorable biomimetic properties, HA has been frequently used as a coating substance [13]. However, HA has been commonly applied to titanium surfaces by plasma spraying, whose data on long-term coating stability are questionable [14,15]. Surface modification strategies such as MP coatings have been evolved to mimic the structural architecture of human bone [7]. However, stability is also a limitation of MP molecules, and polyphosphates are quickly degraded and removed from the coated implant surface. Thus, a monolayer of MP molecules was developed to permanently attach to the implant surface (Nano Bridging Molecules, Gland, Switzerland). 

Several in vitro studies have been performed to elucidate underlying mechanisms and understand interactions between cells and MP-coated implant surfaces. Tîlmaciu et al. demonstrated that surface modification with a phosphonate monolayer on titanium did not impair attachment and proliferation of MC3T3-E1 preosteoblasts [16]. In human osteoblast-like cells, surface functionalization of titanium discs with phosphonates enhanced alkaline phosphatase activity, suggesting beneficial effects on osteoblast maturation in vitro [17]. However, the initial cellular response to MP-coated titanium surfaces regarding osteogenic properties is still poorly understood.

Osteoblasts are directly involved in bone formation and play a crucial role in the osseointegration of dental implants [18,19]. Several cell types were used to investigate the potential impact of various surfaces on osteogenesis. Particularly, MG-63 human osteoblast-like cells have frequently been used in in vitro research investigating implant surfaces. This well-characterized cell line exhibits many osteoblastic traits that are characteristic of bone-forming cells [20,21]. Moreover, bone marrow mesenchymal stem cells (BM-MSCs) and primary human periodontal ligament stem cells (hPDLSCs) can also differentiate into osteoblasts and thus play a direct role in the process of osseointegration [22]. Further, BM-MSCs and osteoblasts have been shown to suppress the activity of osteoclasts that promote bone resorption, dependent on the implant surface properties [23]. In this context, titanium has been demonstrated to be favorable for osteogenic proliferation and differentiation of BM-MSCs compared to other surface materials [24].

Coating of titanium surface might represent a promising strategy to improve the clinical output of titanium devices. Despite promising preclinical and clinical data, knowledge of the influence of MP coatings on osteogenic potential is limited so far. Therefore, the present study aimed to assess the impact of titanium surfaces coated with MP on the expression of osteogenesis-related factors of MG-63 osteoblast-like cells, BM-MSCs, and hPDLSCs in vitro. 

## 2. Materials and Methods 

### 2.1. Titanium Discs Characteristics

Three different moderately-rough titanium grade 3 disk surfaces of 15 × 2 mm were prepared according to proprietary protocols of MIS Implants (Bar-Lev Industrial Park, Israel). MP-coated surfaces underwent common sandblasting and acid etching, after which a monolayer of MP molecules was covalently bound to the titanium surface. The reference surfaces R1 and R2 were sandblasted with medical-grade alumina followed by acid etching at a lower (R1) and a higher (R2) temperature. 

Surface roughness characteristics were determined by optical non-contact profilometry, with an Infinate Focus (Alicona Imaging, Raaba/Graz, Austria) on a 250 × 250 µm field with a Gaussian filter λc of 50 µm [25]. The measurements were performed on 3 areas of 3 different discs. Surface parameters, including the mean arithmetic deviation roughness (Sa), the maximum height of selected area (Sz), skewness of the height distribution (Ssk), and contact angle (θ_CA_) were assessed. The topographic features of the surface of the implants were observed with a VEGA3 SEM (TESCAN, Kohoutovice, Czech Republic) at 20 kV in the secondary electrons (SE) mode; magnification varied between ×800 and ×2000.

### 2.2. Cell Culture

Cultivation of cells was performed for 48 and 120 h, and cells between the third and the sixth passage were used for experiments. The following cell types were grown on the Ti disks and tissue culture plastic (TCP) as control:

#### 2.2.1. MG-63 Osteoblast-Like Cells

MG-63 osteoblast-like cells (American Type Culture Collection, Rockville, MD, USA) were cultivated in minimum essential medium patterned after Eagle’s Medium (MEM, Gibco, Carlsbad, USA), with an additional component of 10% fetal bovine serum, penicillin (100 U/mL), and streptomycin (50 µg/mL) at 37 °C with 5% CO_2_ in a humidified chamber. MG-63 cell culture was performed at a density of 2 × 10^5^ cells/well in 0.5 mL of MEM in 24 well plates and grown for 48 h and 120 h. 

#### 2.2.2. Human Periodontal Ligament Stem Cells (hPDLSCs) 

Wisdom teeth from 3 different patients with a healthy periodontium were removed before orthodontic treatment and utilized to isolate primary hPDLSCs, as specified previously [26]. The patient’s written consent was obtained before the surgical procedure. The Ethics Committee affiliated to the Medical University of Vienna approved the use of cells for this study purpose (ethical approval number: 1694/2015, renewed in 2019). All procedures followed the “Good Scientific Practice” regulations of the Medical University of Vienna and the ethical principles stated by the Declaration of Helsinki. Primary hPDLSCs were cultured in 24 well plates at a density of 2 × 10^5^ cells/well in 0.5 mL of Dulbecco’s modified Eagle’s medium (DMEM, Sigma-Aldrich, St. Louis, MO, USA) with an additional component of 10% fetal bovine serum (FBS, Gibco, Carlsbad, USA), 1% penicillin, and streptomycin (P/S, Gibco, Carlsbad, CA, USA) under humidified conditions.

#### 2.2.3. Bone Marrow Mesenchymal Stem Cells (BM-MSC)

Bone-marrow-derived mesenchymal stem cells (ATCC^®^ PCS-500-012^™^) were cultivated in 24 well plates at a density of 2 × 10^5^ cells/well in 0.5 mL of mesenchymal stem cell basal medium for adipose, umbilical, and bone-marrow-derived MSCs (ATCC^®^ PCS-500-030^™^) under humidified conditions.

### 2.3. Fluorescence Microscopy

After 2 days of culture of the MG-63 cells, hPDLSCs, and BM-MSCs at a density of 10^4^ cells/well, cell staining using an actin cytoskeleton and focal adhesion staining kit (Catalog Number FAK100, Millipore, Burlington, MA, USA) was conducted following the instructions provided by the manufacturer. Microscopy was performed with a fluorescent microscope (Revolve4 RVL-100-G, Echo, San Diego, CA, USA) at different magnifications (10×, 20×, 40×). Cell culture time considerations were based on a previous study investigating cell attachment by fluorescence microscopy [27] and aimed to detect only the initial attachment of cells to Ti surfaces.

### 2.4. Cell Proliferation/Viability

Cell proliferation was assessed using the Cell Counting Kit-8 (CCK-8). WST-8 (2-(2-methoxy-4-nitrophenyl)-3-(4-nitrophenyl)-5-(2,4-disulfophenyl)-2H-tetrazolium, monosodium salt) is a highly water-soluble tetrazolium salt and is reduced by dehydrogenase activities in cells to give a yellow-color formazan dye that is soluble in the tissue culture medium. The quantity of formazan dye produced by dehydrogenase activity in cells directly correlates with the cell count. The MG-63 osteoblast-like cells, hPDLSCs, and BM-MSCs were incubated with the different Ti surfaces and TCP controls for 48 and 120 h. Afterwards, 50 µL of CCK-8 (Cell Counting Kit-8; Dojindo, Japan) were applied to each well, followed by incubation at 37 °C for 4 h. An ELISA reader (Molecular Devices, Silicon Valley, CA, USA) was used to assess cell growth rate by determining the optical density (OD) at 450 nm.

### 2.5. Quantitative Real-Time PCR

Gene expression of markers for osteogenesis and bone turnover in the MG-63 cells, BM-MSCs, and hPDLSCs was determined by quantitative PCR as described elsewhere [26]. The production of cell lysates was performed using the TaqMan Gene Expression Cells-to-CT^TM^ kit (Ambion/Applied Biosystems, Foster City, CA, USA) according to the manufacturer’s instructions [28]. qPCR was conducted using an ABI StepOnePlus device (Applied Biosystems) in paired reactions applying the Taqman gene expression assays with ID numbers as follows (all from Applied Biosystems): collagen 1, Hs00164004_m1; alkaline phosphatase (ALP), Hs01029141_g1; osteoprotegerin (OPG), Hs00171068_m1; osteocalcin (OC), Hs00609452_g1;; RANKL Hs00243522_m1, β-actin, Hs99999903_m1 that was utilized as a house keeping gene. The PCR reactions were done in triplicate, using the following thermocycling conditions: 95 °C for 10 min; 50 cycles, each for 15 s at 95 °C and at 60 °C for 1 min. The point at which the PCR product was first detected above a fixed threshold (cycle threshold, Ct) was determined for each sample. Variations in the production of target genes were calculated using the 2-DDCt method, where DDCt = (Ct^target^ − Ct ^β-actin^)_sample_ − (Ct ^target^ − Ct ^β-actin^)_control_, at which cells cultured on tissue culture plastic (TCP) were utilized as a control.

### 2.6. Statistical Analysis

Normal data distribution was tested with the Kolmogorov–Smirnov test. An analysis of statistical difference between the impact of the different titanium surfaces on the cultivated cells was performed by one-way analysis of variance (ANOVA) for repeated measures followed by the Student *t*-test. All statistical calculations were conducted using SPSS (Statistics Software v 24.0; IBM Corp, Armonk, NY, USA). A *p*-value <0.05 was regarded to be statistically significant. Data are expressed as mean ± standard error of the mean (S.E.M.). Repetition of experiments was performed at least three times. 

## 3. Results

### 3.1. Surface Characteristics

The following surface characteristics were assessed: MP-coated surfaces (Sa = 0.87 ± 0.08 µm, Sz = 11.25 ± 1.38 µm, Ssk = −0.19 ± 0.17 µm, θ_CA_ = 100.71°), R1 (Sa = 0.88 ± 0.09 µm, Sz = 11.00 ± 1.96 µm, Ssk = -0.15 ± 0.15 µm, θ_CA_ = 99.5°), R2 (Sa = 1.04 ± 0.07 µm, Sz = 12.16 ± 1.55 µm, Ssk= −0.05 ± 0.13 µm, θ_CA_ = 105.87°). The Sa values of MP-coated and R1 surfaces were significantly lower (*p* < 0.01) than those of the R2 surfaces. No significant differences in Sz, Ssk, and θ_CA_ were detected between different surfaces.

### 3.2. Fluorescence Microscopy

**Figure 1:**
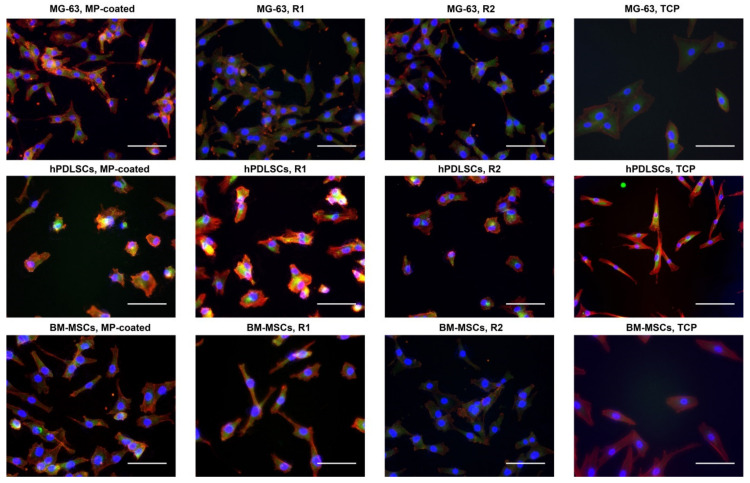
Fluorescence microscopy images of MG-63 cells, hPDLSCs, and BM-MSCs cultured on titanium surfaces and tissue culture plastic (TCP). Cell culture was performed on multi-phosphonate (MP)-coated as well as sand-blasted and acid-etched reference surfaces (R1, R2) and tissue culture plastic (TCP) as control for 48 h; F-actin was stained with TRITC-conjugated Phalloidin (**red**), focal adhesions with anti-Vinculin visualized by fluoresceinisothiocyanat (FITC) (**green**) and the nucleus with 4′,6-Diamidin-2-phenylindol (DAPI) (**blue**). Scale bars correspond to 100 µm.

Cell attachment to the different surfaces after 48 h of culture is shown in Figure 1 and Supplementary Figures S1–S3. Within each cell type, no differences in cell morphology were observed between all tested Ti surfaces. However, hPDLSCs and BM-MSCs exhibited a more pronounced spindle-like shape on the TCP compared to all Ti surfaces.

### 3.3. Cell Proliferation/Viability

**Figure 2:**
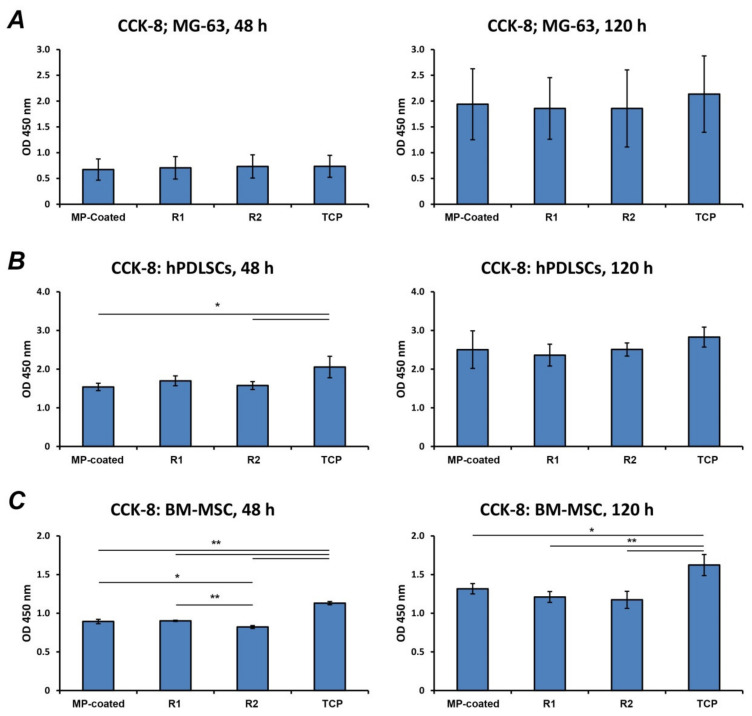
Proliferation/viability of different cells cultured on titanium surfaces and TCP.MG-63 cells (**A**), human periodontal ligament stem cells (hPDLSCs) (**B**), and bone marrow mesenchymal stem cells (BM-MSCs) (**C**) were cultured on multi-phosphonate (MP)-coated as well as sand-blasted and acid-etched reference surfaces (R1, R2), and cell proliferation/viability was assessed after 48 and 120 h by Cell Counting Kit-8 (CCK-8) experiments, which are based on the measurements of cell metabolic activity. Cells cultivated on tissue culture plastic (TCP) served as control. The Y-axis corresponds to the optical density (OD) values assessed at 450 nm. Data are presented as mean ± standard error of the mean (S.E.M) of three experiments that were conducted independently. * and **—significantly different between groups with *p* < 0.05 and *p* < 0.01, respectively.

Cell proliferation/viability of various cell types grown on different Ti surfaces is presented in Figure 2. The proliferation/viability of MG-63 was similar in all investigated surfaces and TCP after both 48 and 120 h of culture (Figure 2A). In hPDLSCs (Figure 2B), the proliferation/viability was similar for all Ti surfaces after both time points. After 48 h, the proliferation/viability of hPDLSCs cultivated on MP-coated (*p* < 0.05) and R2 (*p* < 0.05) surfaces was significantly decreased compared to TCP, whereas no significant differences were observed after 120 h. Cell proliferation/viability of BM-MSCs (Figure 2C) at 48 h was significantly increased on TCP in comparison to all test surfaces (*p* < 0.01), and proliferation on R2 was lower than MP-coated (*p* < 0.05) and R1 (*p* < 0.01) surfaces. At 120 h, proliferation on TCP was significantly higher compared to other surfaces (MP-coated *p* < 0.05 vs. MP-coated; *p* < 0.01 vs. R1 and R2).

### 3.4. Gene Expression in MG-63 Cells

**Figure 3:**
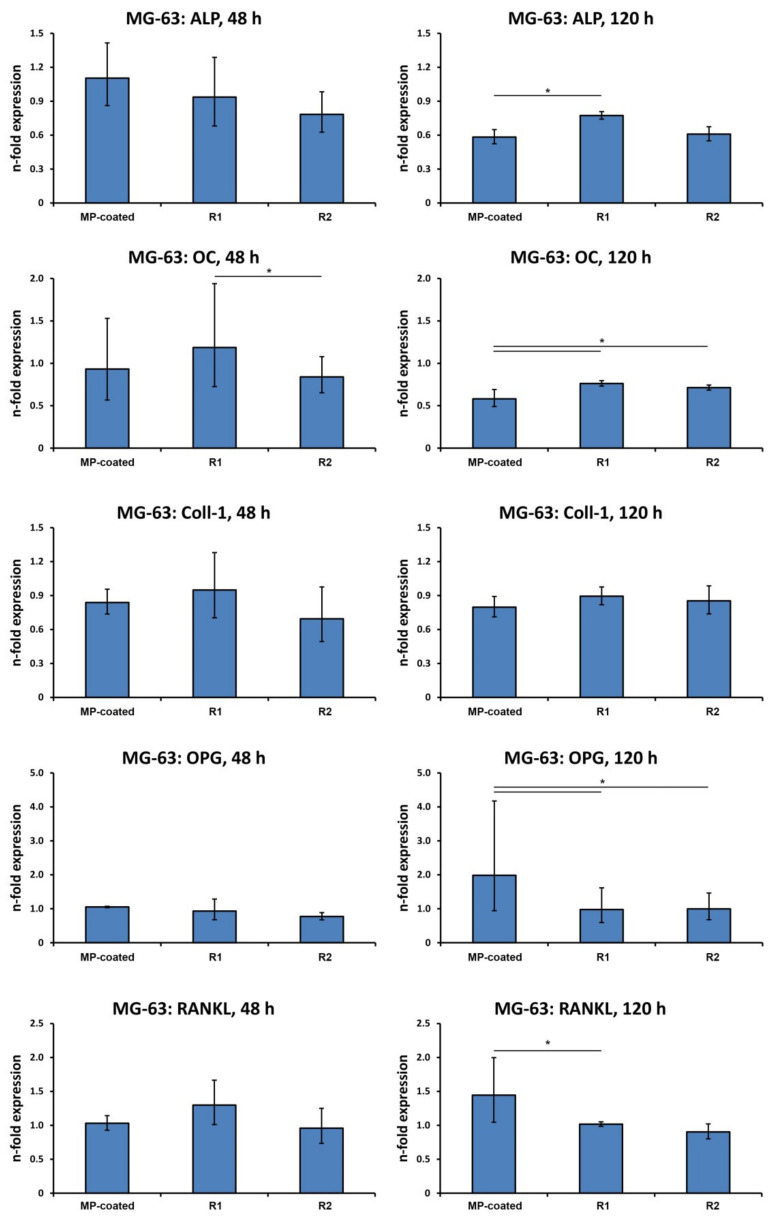
Expression of genes related to osteogenesis and bone turnover in MG-63 cells grown on titanium surfaces.MG-63 cells were cultivated on multi-phosphonate (MP)-coated, sand-blasted, and acid-etched reference surfaces (R1, R2) and tissue culture plastic (TCP) for 48 and 120 h, and gene expression of alkaline phosphatase ALP, osteocalcin OC, collagen type I (Col1), osteoprotegerin (OPG), and receptor activator of NF-κB ligand (RANKL) was assessed by quantitative real-time PCR. Y-axis represents n-fold expression related to the MG-63 cells cultivated on TCP calculated using the 2^−ΔΔCt^ method, using glyceraldehyde 3-phosphate dehydrogenase (GAPDH) as a housekeeping gene. Data are presented as the mean ± S.E.M of four experiments that were conducted independently *—significantly different between groups, *p* < 0.05.

The gene expression levels of different proteins related to osteogenesis and bone turnover in MG-63 osteoblast-like cells grown on different surfaces are shown in Figure 3. After 48 h, the expression of osteocalcin (OC) was significantly lower on R2 compared to R1 (*p* < 0.05). After 120 h, the expression of osteoprotegerin (OPG) and receptor activator of NF-κB ligand RANKL was significantly higher in cells cultivated on MP-coated surfaces compared to R1 disks (*p* < 0.05). Levels of OPG expression were also increased in the MP-coated group compared to R2 (*p* < 0.05). The expression of early and late osteoblast differentiation markers alkaline phosphatase (ALP) and OC was significantly lower on MP-coated surfaces compared to R1 (*p* < 0.05), and OC gene expression was also lower on MP-coated surfaces compared to R2 (*p* < 0.05). No difference in Col1 expression between MP, R1, and R2 was detected.

### 3.5. Gene Expression in hPDLSCs

**Figure 4:**
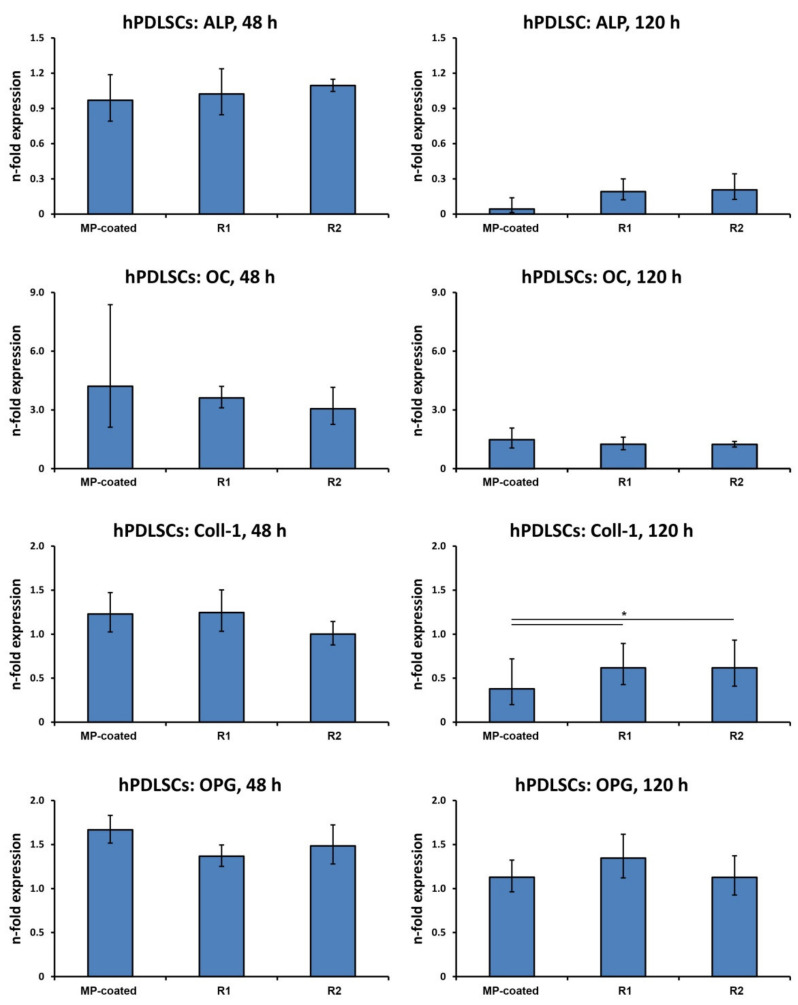
Expression of genes related to osteogenesis and bone turnover in hPDLSCs grown on titanium surfaces. hPDLSCs were cultured on multi-phosphonate (MP)-coated, sand-blasted, and acid-etched reference surfaces (R1, R2) and tissue culture plastic (TCP) for 48 and 120 h, and the expression of ALP, OC, Col1, OPG, and RANKL was assessed by quantitative real-time PCR. Y-axis represents n-fold expression related to hPDLSCs on TCP determined using the 2^−ΔΔCt^ method, using GAPDH as a housekeeping gene. Data are shown as the mean ± S.E.M of four experiments that were conducted independently. *—significantly different between groups, *p* < 0.05.

Figure 4 shows the gene expression of osteogenesis-related proteins and OPG in hPDLSCs after a different culture time. After 48 h, no significant differences in the production of all investigated parameters were determined between the different surfaces. After 120 h, Col1 expression in primary hPDLSCs on the MP-coated surface was significantly lower than on the reference surfaces R1 and R2 (*p* < 0.05), but no differences in the expression of ALP, OC, and OPG was observed. The expression of RANKL in hPDLSCs was below the detection limit of qPCR. 

### 3.6. Gene Expression in BM-MSCs

**Figure 5:**
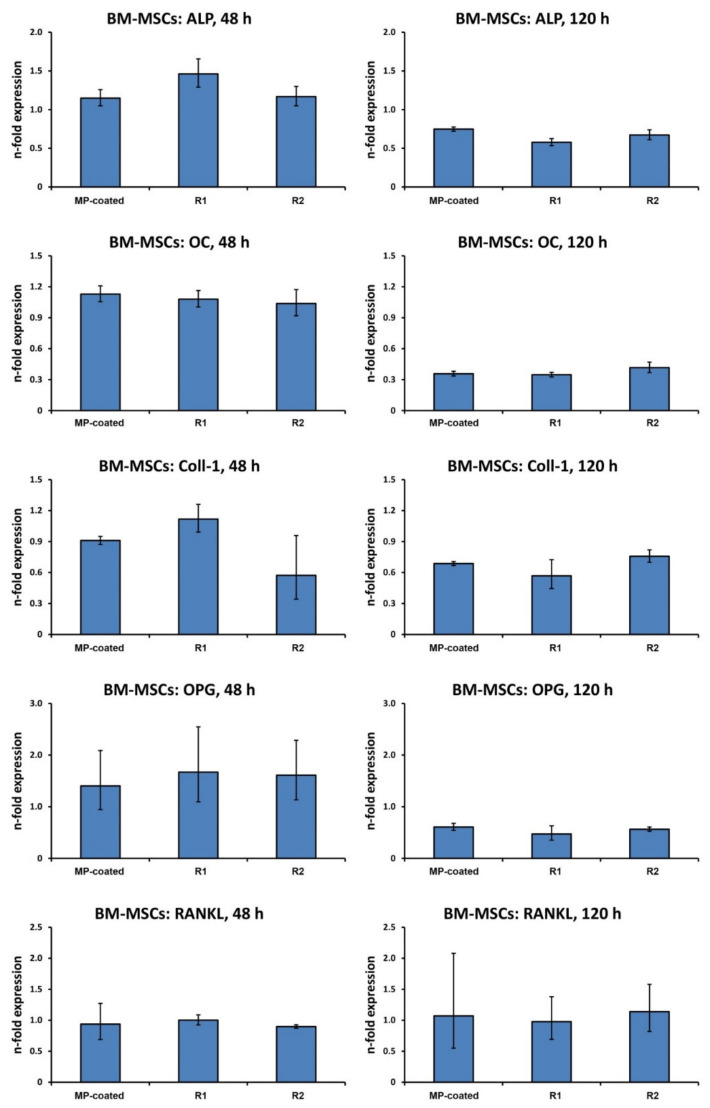
Expression of genes related to osteogenesis and bone turnover in BM-MSCs grown on titanium surfaces. BM-MSCs were cultured on multi-phosphonate (MP)-coated, sand-blasted, and acid-etched reference surfaces (R1, R2) and tissue culture plastic (TCP) for 48 and 120 h, and the expression of ALP, OC, Col1, OPG, and RANKL was assessed by quantitative real-time PCR. Y-axis represents n-fold expression related to BM-MSCs on TCP determined using the 2^−ΔΔCt^ method, using GAPDH as a housekeeping gene. Data are shown as the mean ± S.E.M of four experiments that were conducted independently.

Gene expression of proteins related to osteogenesis bone turnover in BM-MSCs grown on different surfaces is shown in Figure 5. No significant difference in the expression of all investigated proteins between test surfaces was observed after both 48 and 120 h 

## 4. Discussion

The concept of dental implant osseointegration has been described first by Brånemark et al. and is defined as a structural and functional connection between the implant surface and newly formed bone [29].To improve osseointegration, various concepts to modify implant surfaces have been developed. In research from other medical fields, surface modifications have been shown to promote tissue regeneration capacity of scaffold materials [30], or to enhance the biocompatibility of substances such as cellulose when applied in the rat model [31]. Research in implant dentistry has focused on changes to the physical and chemical qualities of titanium surfaces, including topography, chemistry, surface charge, and hydrophilicity, to develop surfaces that provide favorable conditions for osseointegration [32]. Among various approaches for surface modifications in dental implantology, coating strategies that mimic the biochemical milieu and nanostructural architecture of human bone have raised interest within the last years [33,34]. Phosphorous containing nanostructured surface coatings establish a direct chemical bond connecting bone and titanium and have been considered a promising approach to improve osseointegration by mimicking natural hydroxyapatite [35,36].

To date, few clinical studies on MP-coated implants have been performed and in vitro data on the effects of this MP coating on osteoblast response are scarce. In a pelvic sheep model, MP coating of titanium implants demonstrated significantly greater biomechanical stability in terms of higher removal torque after 52 weeks; this was supported by an SEM analysis suggesting a strong bond between the surface of implants that had undergone MP treatment and the adjacent osseous tissue [6]. In a split-mouth designed study on 23 patients, the 1-year survival rate of MP-coated implants has been 100%, with a comparable mean marginal bone level change between the test and control groups [7]. A three-year follow-up of the same cohort showed no statistically significant differences regarding marginal bone level alteration between MP-coated implants and untreated control; three patients dropped out, and complications were reported for another three patients concerning both implant types [37]. 

To shed light on the potential effect of MP coating on osteogenesis, we focused on the evaluation of osteogenesis-related markers, such as alkaline phosphatase (ALP), collagen type I (Col1), and osteocalcin (OC), have been frequently used to assess osteogenic differentiation potential in vitro. ALP and Col1 are markers for early osteoblast differentiation [38,39], whereas OC is considered a late differentiation marker [40]. Moreover, bone resorption and formation are coupled by interactions between osteoprotegerin (OPG) and receptor activator of NF-κB ligand (RANKL). RANKL is known to activate bone osteoclasts that promote bone resorption. OPG is a decoy receptor binding to RANKL and thereby inhibiting its capability to stimulate osteoclasts. Therefore, the OPG/RANKL system is critical for controlling bone turnover [41]. 

According to the present findings, cell morphology of MG-63 osteoblast-like cells showed only slight differences between titanium surfaces and plastic control, which is in accordance with a previous study using human osteoblasts [42]. Various cell types exhibited similar proliferation/viability when exposed to MP-coated titanium surfaces, although some differences were observed on the gene level. In MG-63 osteoblast-like cells, both bone turnover markers, OPG and RANKL, were increased on the MP-coated surface, while OPG expression levels were more enhanced than those of RANKL. This suggests a higher bone turnover rate in favor of bone formation. Likewise, MG-63 cells have shown similar results when exposed to hydroxyapatite and dicalcium silicate particles that are used as an implant surface coating [43]. The favorable effect of MP coating on bone formation by affecting the OPG/RANKL system should be further investigated by animal or clinical studies. 

In contrast, MG-63 osteoblast-like cells exhibited lower expression levels of osteogenesis-related proteins when cultured on the MP-coated surface. The level of ALP expression was higher on R1 surfaces compared to disks with MP coating. This finding is partly confirmed by Borsari et al., who demonstrated a decrease of ALP activity and collagen type I protein production in MG-63 cells cultured on hydroxyapatite-coated titanium surfaces; however, the gene expression level of ALP was not determined in the respective study [44]. Moreover, according to the present results, OC expression on MP-coated surfaces was significantly lower than on the reference surfaces. This is in contrast to a previous study by Filová et al., who showed that the protein production of OC in MG-63 osteoblast-like cells increased with the concentration of micro-sized HA particles when cultured on HA composite surfaces [45]. When comparing to the present study, it has to be taken into account that cells were cultured on a composite material based on polyamide balanced fabric reinforcement, not titanium, and gene expression was not investigated. Furthermore, slight differences in cell response between the three cell types used in the present study could partly be explained by the fact that each cell type has distinct characteristics. MG-63 is an osteosarcoma cell line, which has constant properties and exhibits osteoblastic features [20,46]. In contrast, primary osteoblasts show a different response that is dependent on the origin, donator, and isolation technique [47,48]. However, it should be noted that the effects of MP coating on the expression of osteogenesis-related genes were rather small, and their physiological relevance needs to be further clarified.

hPDLSCs have the potential to play a decisive role during osseointegration for implants installed into fresh extraction sockets and have been demonstrated to be highly proliferative and multipotent [49]. hPDLSCs have osteogenic, adipogenic, and chondrogenic differentiation potential [50]; they have been demonstrated to proliferate and show signs of osteogenic differentiation on titanium surfaces [51]. A previous study by Marconi et al. confirmed the present findings that surface treatment did not have an essential impact on the morphology of hPDLSCs grown on titanium surfaces [52]; however the study did not investigate plastic control. According to the present study, a lower Col1 gene expression in hPDLSCs after 120 h was detected on the MP-coated surface compared to the reference samples, which could also hint at reduced osteogenesis under in vitro conditions. In a study by Heo et al., Col1 gene expression was reduced after six days, which is in line with the present findings [51]. Similar to MG-63 cells, the physiological relevance of the observed effects of MP-coating on osteogenesis in hPDLSCs is questionable and should be investigated by further studies.

BM-MSCs are pluripotent cells capable of differentiating towards osteoblastic lineage, and are recruited from bone marrow during the osseointegration process [22]. According to the present fluorescence microscopy images, cell morphology of BM-MSCs was similar between titanium surfaces, however a more flattened and extended shape was observed on TCP. MSCs’ morphology is considered to be an important parameter affecting cell differentiation fate independently of other factors [53]. In general, literature on cell morphology of BM-MSCs cultivated on titanium surfaces is limited. Although a previous study by Colombo et al. investigated cell shape and adhesion [54], it has to be taken into account that in the respective study, the evaluated surfaces exhibited both higher and lower Ra levels than our samples, and TCP was not investigated. Interestingly, the expression of all investigated genes in the present study was similar on all test surfaces in BM-MSCs, suggesting no impact of the MP coating. This is not in line with the findings of Lin et al., according to which hydroxyapatite induced an increase of osteocalcin in BM-MSCs [55]. The discrepancy could be partly explained by the cell type used. In contrast to Lin et al., who performed experiments with ***C***3H10T1/2, pluripotent mouse stem cells, we used mesenchymal stem cells from human origin. In another study, BM-MSCs showed a higher expression of ALP, RUNX2, and OC expression upon cultivation on titanium surfaces with increased hydrophilicity, suggesting an enhanced differentiation towards osteoblastic lineage [56]. According to the present findings, no significant differences in osteogenic response in BM-MSCs upon cultivation on MP-coated surfaces were observed. However, this might be due to different surface modification procedures, possibly influencing osteogenic cell response through a diverse mechanism.

In the present study, the R2 surface was slightly but significantly rougher than the MP-coated and R1 surfaces. These differences could be explained by some differences in the etching procedure. MP-coated and R1 surfaces were etched at a lower temperature than the R2 surface. Increasing the temperature intensifies the strength of the attack of the etching acid bath and therefore establishes a rougher surface. Thus, the R2 surface was within the range of moderate roughness (Sa = 1.04 ± 0.07 µm) as defined by Wennerberg et al., and the other two were minimally rough (R1: Sa = 0.88 ± 0.09 µm; MP surface: Sa = 0.87 ± 0.08 µm) [57]. However, the differences between the Sa values seemed to be too small to be relevant with regard to the evaluated parameters. Particularly, our previous study showed that the increase of Ra from 0 to 1 has a strong effect on the expression of osteogenesis-related factors in osteoblasts, whereas further increases or Ra up to 2 µm have a rather small effect on these characteristics [21]. 

One limitation of this study is that experiments were restricted to the gene-level investigation. We did not investigate the effect of MP coating on osteogenic differentiation in specific osteogenic media. Although no substantial impact is expected, the experimental data still has to confirm or reject its existence. As another study limitation, it has to be stated that osteogenic differentiation was not assessed, e.g., by alizarin red staining to visualize matrix calcification. Furthermore, valuation of toxicity or inflammation would be required and should, therefore, be addressed in further studies to assess the MP coating’s biocompatibility. The potential of MP coating to improve bone turnover through the influence of the OPG/RANKL system should be further confirmed by animal and/or clinical studies.

## 5. Conclusions

Our findings suggest that the MP coating did not increase cell proliferation/viability in vitro and had no beneficial effects on osteogenic differentiation. 

## Supplementary Materials

The following are available online at https://www.mdpi.com/1996-1944/13/24/5777/s1, Figure S1: Fluorescence microscopy analysis of focal adhesions in MG-63 cells, hPDLSCs, and BM-MSCs cultured on titanium surfaces and TCP, Figure S2: Fluorescence microscopy analysis of F–actin in MG-63 cells, hPDLSCs, and BM-MSCs cultured on titanium surfaces and TCP, Figure S3: Fluorescence microscopy analysis of nuclei in MG-63 cells, hPDLSCs, and BM-MSCs cultured on titanium surfaces and TCP.
