# Supplementary material for: Effect of Multi-Phosphonate Coating of Titanium Surfaces on Osteogenic Potential
Source: Materials (Basel). 2020 Dec 17;13(24):5777. doi: 10.3390/ma13245777 (PMC7766650; doi:10.3390/ma13245777)
Supplement: Supplementary file 1 [file materials-13-05777-s001.zip › materials-1010622-supplementary.pdf]

materials

MDPI

Article

Effect of Multi-Phosphonate Coating of Titanium Surfaces on Osteogenic Potential

Christian Wehner [1], Christian Behm [2,3], Selma Husejnagic [1], Andreas Moritz [1], Xiaohui Rausch-Fan [1,2] and Oleh Andrukhov [2,*]

[1] Division of Conservative Dentistry and Periodontology, University Clinic of Dentistry, Medical University of Vienna, 1090 Vienna, Austria; christian.wehner@meduniwien.ac.at (C.W.); selma.husejnagic@meduniwien.ac.at (S.H.); andreas.moritz@meduniwien.ac.at (A.M.); xiaohui.rausch-fan@meduniwien.ac.at (X.R.-F.)

[2] Competence Center for Periodontal Research, University Clinic of Dentistry, Medical University of Vienna, 1090 Vienna, Austria; christian.behm@meduniwien.ac.at

[3] Division of Orthodontics, University Clinic of Dentistry, Medical University of Vienna, 1090 Vienna, Austria

* Correspondence: oleh.andrukhov@meduniwien.ac.at; Tel.: +43-1-40070-2620

Received: 6 November 2020; Accepted: 15 December 2020; Published: 17 December 2020

Supplementary Information

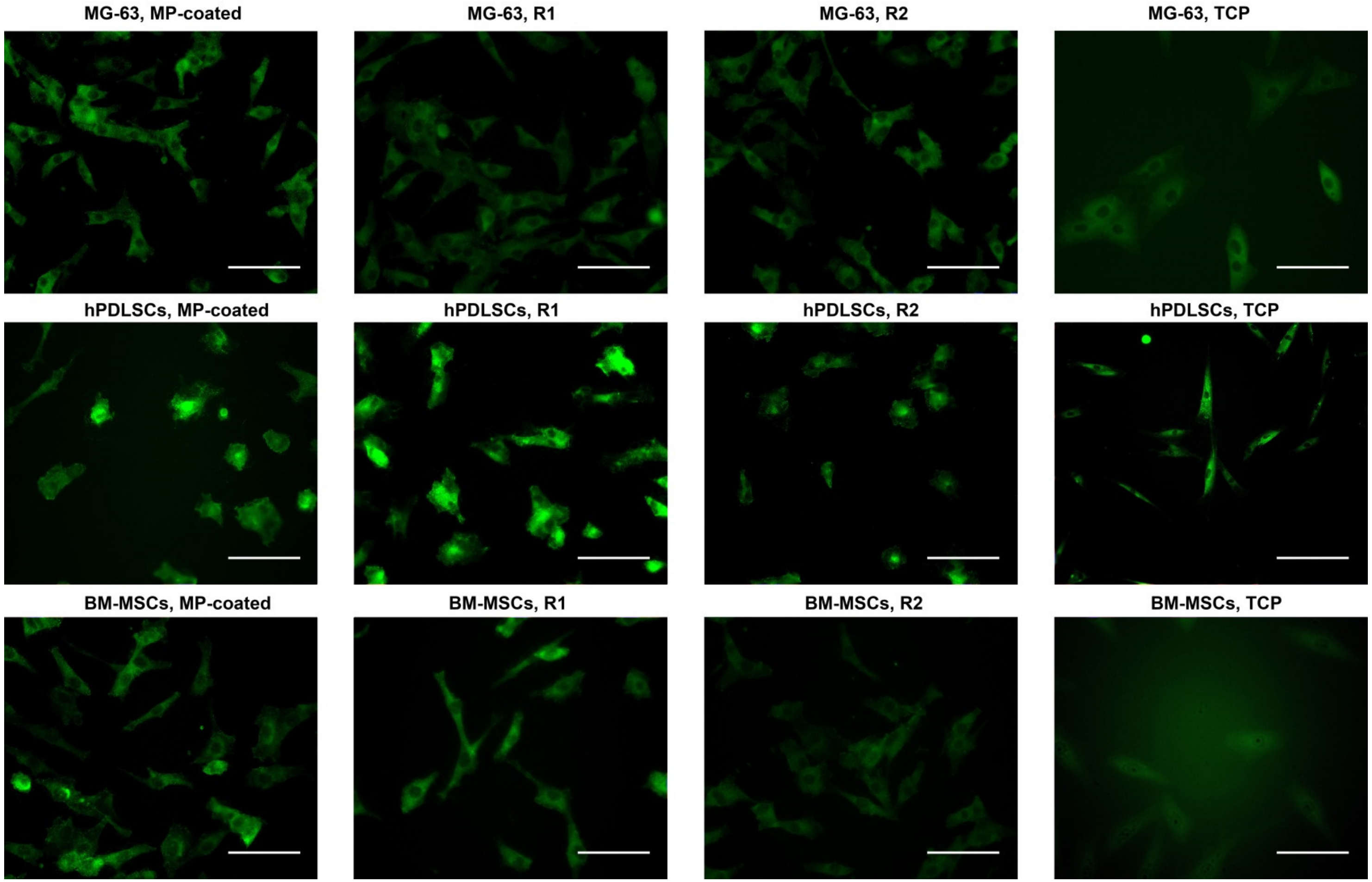

Supplementary Figure S1. Fluorescence microscopy analysis of focal adhesions in MG-63 cells, hPDLSCs, and BM-MSCs cultured on titanium surfaces and TCP.

Cell culture was performed on MP-coated as well as reference surfaces (R1, R2) and TCP as a control for 1 and 2 days; focal adhesions were stained with anti-Vinculin and counterstained with FITC (green). Scale bars correspond to 100 μm.

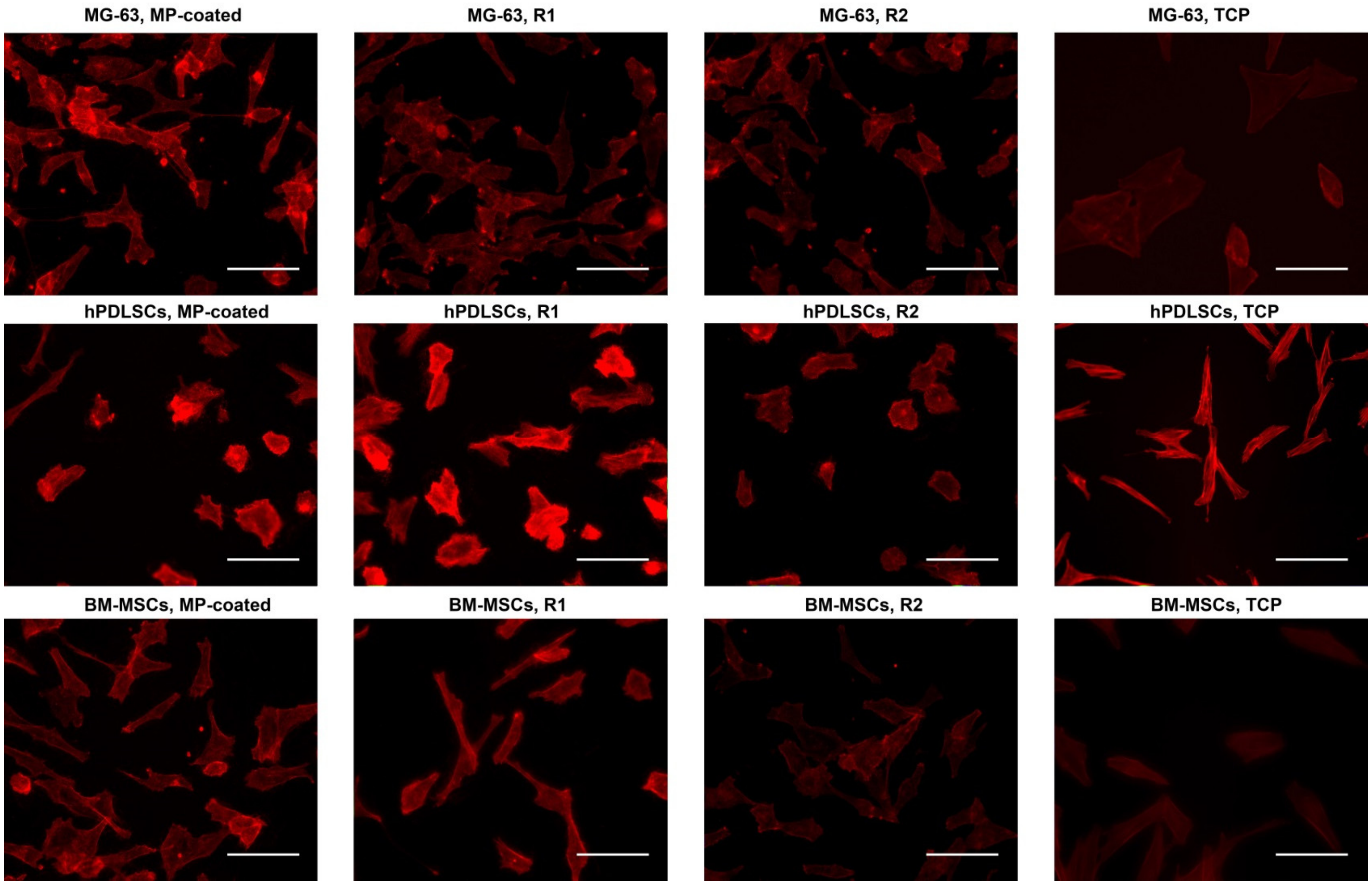

Supplementary Figure S2. Fluorescence microscopy analysis of F–actin in MG-63 cells, hPDLSCs, and BM-MSCs cultured on titanium surfaces and TCP.

Cell culture was performed on MP-coated as well as reference surfaces (R1, R2) and TCP as a control for 1 and 2 days; F-actin was stained with TRITC-conjugated Phalloidin (red). Scale bars correspond to 100 µm.

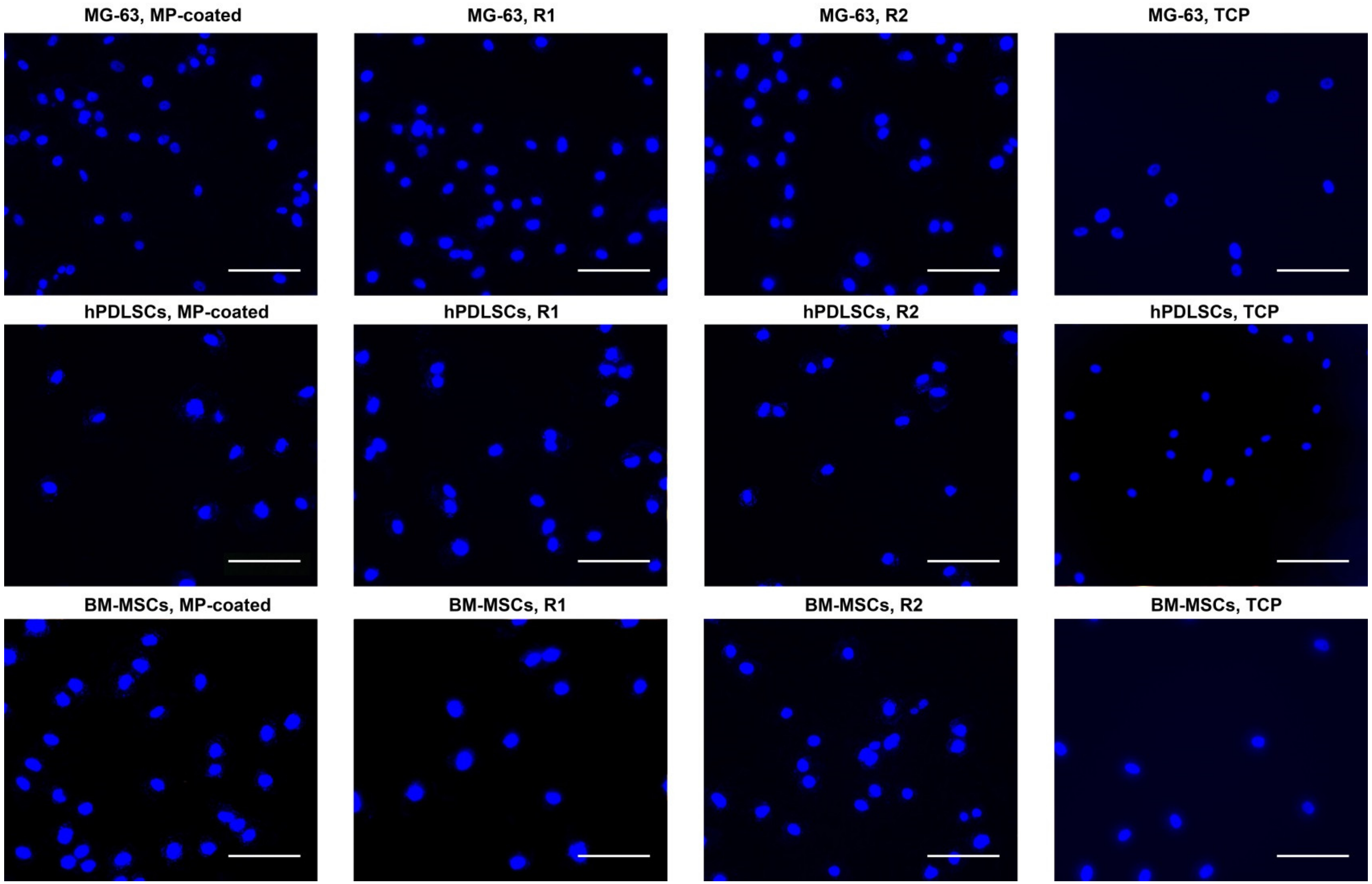

Supplementary Figure S3. Fluorescence microscopy analysis of nuclei in MG-63 cells, hPDLSCs, and BM-MSCs cultured on titanium surfaces and TCP.

Cell culture was performed on MP-coated as well as reference surfaces (R1, R2) and TCP as a control for 1 and 2 days; nuclei were stained with DAPI (blue). Scale bars correspond to 100 µm.

Publisher's Note: MDPI stays neutral with regard to jurisdictional claims in published maps and institutional affiliations.

© 2020 by the authors. Licensee MDPI, Basel, Switzerland. This article is an open access article distributed under the terms and conditions of the Creative Commons Attribution (CC BY) license (http://creativecommons.org/licenses/by/4.0/).
